# Bridging the Gap between Authentic Leadership and Employees Communal Relationships through Trust

**DOI:** 10.3390/ijerph17010250

**Published:** 2019-12-30

**Authors:** Sadaf Iqbal, Tahir Farid, Muhammad Khalil Khan, Qionghon Zhang, Amira Khattak, Jianhong Ma

**Affiliations:** 1 Department of Applied Psychology and Behavioral Science, Zhejiang University, 866 Yuhangtang Road, Hangzhou 310058, China; sadaf@zju.edu.cn (S.I.); tahir_khattak@zju.edu.cn (T.F.); joannazhang@zju.edu.cn (Q.Z.); 2 College of Media and International Culture, Zhejiang University, 866 Yuhangtang Road, Hangzhou 310058, China; khan@zju.edu.cn; 3 Department of Marketing, Prince Sultan University, P.O. Box 66833, Riyadh 11586, Saudi Arabia; akhattak@psu.edu.sa

**Keywords:** authentic leadership, affective-based trust, cognitive-based trust, communal relationship, banking sector, Pakistan

## Abstract

Authentic leadership has emerged as a positive relational-leadership approach that has gained the attention of academicians and practitioners by stimulating a healthy work environment. This study examined the direct influence of authentic leadership on employees’ communal relationships. In addition, the study examined the mediating role of affective- and cognitive-based trust on these relationships. We adopted a cross-sectional study design and collected data from 200 employees working in the private banking sector in Pakistan. The findings indicated that authentic leadership was positively correlated with communal employee relationships. In addition, both affective- and cognitive-based trust were found to have a positive mediating effect on the relationship between authentic leadership and communal employee relationships. The practical implications, limitations and suggestions for future research are discussed.

## 1. Introduction

Employees’ positive organizational relationships not only play an important role in shaping organizational reputation, but also contribute towards the achievement of organizational aims and boosting employee effectiveness. Likewise, no assignment can be effectively achieved if disagreement occurs between employees, and their organization [1,2]. Employees who maintain a respectable relationship with their leadership and the organization in which they are working consider organizational problems as their own and try to find solutions for them as if they were their own problems [3]. Employees’ communal relationships are a form of positive relationship, introduced by Clark and Mills [4] as “the efforts to give benefits to other party with no expectation of returns in future”. Empirical evidence reveals that a communal relationship plays a significant role in assisting organizational management to pay specific attention to its duties because it then assists its employees without any expectations [5,6].

Authentic leadership has nowadays appeared as a form of leadership style that has gained the attention of numerous scholars [7,8,9] and practitioners [10,11,12]. The interest on authentic leadership stems from the recent corporate scandals and organizational malfeasance, like leadership dishonesty and unethical practices [13], and the reduction of conventional leadership approaches, such as transformational and charismatic leadership, which emphasize attaining organizational performance through leadership. Centering on authenticity and morality, authentic leadership focuses on ethical dilemmas and motivates firms to establish a positive learning and organizational climate [14,15]. In addition, authentic leadership has attracted researchers’ attention due to its positive influence on employees’ job outcomes and organizational-goal achievements [16,17,18], and the call for more empirical work [7,8,19].

Empirical evidence reveals the significant role of authentic leadership in affecting employees’ workplace outcomes [20]. For instance, authentic leadership has been revealed to enhance employees’ organizational citizenship behavior (OCB) [16,17,21] positively related to an ethical culture [22], improving employees’ organizational commitment [23], increasing employees’ work engagement [24,25], better employee performance [26], and trust [25,27]. However, little research exists into investigating the direct impact of authentic leadership and employees’ communal relationships [9]. In addition, although empirical evidence reveals a positive association between authentic leadership and employees’ communal relationships [9], the psychological mechanism underlying this relationship is less clear. 

This research makes an important addition to the literature on authentic leadership by examining a novel mediation framework that explains the procedure through which authentic leaders impact their subordinates’ communal relationships. Focusing on relational-model theory [28] and social-exchange theory [29], we examined the mediating effect of affective- and cognitive-based trust in transmitting the effect of authentic leadership to enhance employees’ communal relationships. Authentic leadership is defined as having optimistic beliefs that inculcate qualities of hope, trust, and positive emotions in their subordinates [7,30,31]. Similarly, Dirks and Ferrin [32], in their meta-analytic review, called for scholars to consider two dimensions of trust, affective- and cognitive-based trust, and to “attempt to distinguish between the processes involved” (p. 623), however, very few studies have followed this call [33,34,35]. In addition, this is the first study that focuses on affective- and cognitive-based trust as a mediating variable while examining the relationship between authentic leadership and employees’ communal relationships. It is suitable to explore the mediating effect of affective- and cognitive-based trust at the individual level, as they were initially theorized as individual-level constructs; an increasing body of research has also revealed leadership’s influence on individuals and group levels [36].

Conducting research related to authentic leadership and OCB in the context of Pakistan is important because most research related to these topics has been conducted in Western cultures, which are quite different from South Asian cultures (specifically Pakistani culture); this is a new contribution by examining this relationship in Pakistan’s collectivistic culture.

In addition, past studies have urged for more empirical research to examine authentic-leadership types globally [7,20]. This study is an attempt to respond to this call, and it explores the role of authentic leadership in affecting employees’ communal relationships by focusing on the collectivistic culture of Pakistan, which is quite different from Western culture. In addition, this is a three-way study in which authentic leadership influences employees’ communal relationship through two sequential mediators, namely, affective- and cognitive-based trust. By investigating processes through which these two dimensions of trust transmit the effects of authentic leadership on employees’ communal relationships, our study extends the authentic-leadership and trust literature in a new important direction.

## 2. Theory and Hypothesis

### 2.1. Authentic Leadership

Authentic leadership (AL) appeared as a significant area of research in the academic arena with the emergence of the positive-psychology movement [13]. According to Avolio, Luthans, and Walumbwa (2004), authentic leaders are those “who are deeply aware of how they think and behave and are perceived by others as being aware of their own and others values/moral perspectives, knowledge and strengths; aware of the context in which they operate; and who are confident, hopeful, optimistic, resilient, and of high moral character” (p.4). The definition of authentic leadership given by Walumbwa, Avolio [16] is the one most commonly used in the academic literature. They define AL as: “a pattern of leader behavior that draws upon and promotes both positive psychological capacities and a positive ethical climate, to foster greater self-awareness, an internalized moral perspective, balanced processing of information, and relational transparency on the part of leaders working with followers, fostering positive self-development” (p. 94).

Walumbwa, Avolio [16] suggested four components of authentic leadership. Self-awareness is related to the awareness of one’s strengths and weaknesses, and their values and beliefs [37]. Authentic leaders have a stable sense of self-knowledge [38]. Relational transparency is related to presenting one’s true self to other people, assisting in forming trust and cooperation, and nurturing teamwork among colleagues [31]. Balanced processing is related to a leader’s ability to be unbiased in considering all relevant information before reaching any decision [39]. Internalized moral perspective is related to the leaders beliefs and moral values that are compatible with their behaviors [16]. Leaders are considered to be authentic when they represent these four components. The theoretical and empirical findings of past studies also recommend that authentic leadership can be the association between the four aforementioned components [16,31,40,41].

### 2.2. Authentic Leadership and Employees’ Communal Relationships

Authenticity plays a significant role in influencing employees’ organizational relationships in the subject of organizational behavior. Employees’ experiences of authentic organizational behavior depend on transparency, trustfulness, and consistency in leadership approaches [42]. In addition, even though many scholars examined the employees’ organizational relationships [43,44], uncertainty still remains. Hon and Grunig [5] emphasized that management must develop communal relationships with their employees in order to enhance the importance of employees’ organizational relationships. Such types of actions could enable management to concentrate on their duties, profiting employees without any hope of returns [5]. Recent empirical evidence revealed the positive role of authentic leadership in influencing employees’ communal relationships [9]. 

**Figure 1:**
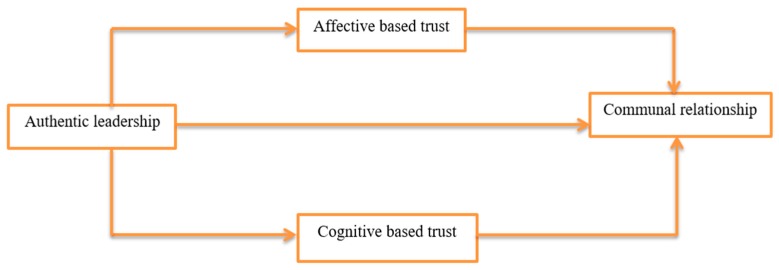
Hypothetical model of relationship between authentic leadership (independent variable), affective- and cognitive-based trust (mediators), and communal relationships (dependent variable).

The association between authentic leadership with employees’ communal relationships can be discussed from the perspective of relational-model theory [28,45], which comprises four major mental schemas: communal sharing, equality matching, authority ranking, and market pricing. Communal-sharing schemas indicate showing care and concern for other people and satisfying their necessities [28]. Similarly, Iqbal, Farid [9] demonstrated that communal-sharing schemas could be linked with authentic leadership, as authentic leaders could increase social interactions with their subordinates, show concern for other members in a neutral manner, and gain precise information before making any decision. Therefore, on the basis of the theoretical component of the communal-sharing mental schema, it was conceived that, when employees perceive their leader’s behavior as authentic, they exhibit more interest in their work and show care for organizational reputation, which helps the organization become more successful. Hence, it is suggested that authentic leadership is positively related with employees’ communal relationships (Figure 1) and the following is proposed:

**Hypothesis** **1.** Authentic leadership is positively correlated with employees’ communal relationships.

### 2.3. Authentic Leadership and Trust 

Trust refers to “a psychological state including the intention to accept vulnerability based upon optimistic expectations of the intentions or behavior of another” [46]. On the basis of the aforementioned definition, we can say that the extent to which employees show their willingness to subject themselves to work, in the interest of their leader, depends upon the exchange relationship between employee and leader. Social-exchange theory [29] also suggests that the actions of individuals depend on the rewards they received from others in the past or expect to receive in the future [47]. Konovsky and Pugh [48] stated that trust was a basic element in developing and fostering exchange-based relationships. In addition, leadership and trust could also be perceived as an exchange relationship between leaders and followers [49]. We chose the two-dimensional trust model of McAllister [50] (affective- and cognitive-based trust) to better understand how affective- and cognitive-based trust mediates the relationship between authentic leadership and employees’ communal relationships. We adopted McAllister’s [50] model of trust for several reasons. First, although past studies revealed a strong association of authentic leadership with trust, previous scholars have typically ignored the multidimensionality of the trust construct [25,27,51,52,53]. Second, in recent years [50], the model of affective- and cognitive-based trust has been profoundly examined and validated in different contexts [27,32,34]. Finally, the McAllister [50] model of trust is more commonly used in leadership studies relative to other existing trust models [35,54,55].

Affective-based trust is a relation-based approach, developed on the basis of ongoing exchanges of social relationships rather than economic exchanges, and it is comprised of care, concern, and mutual obligation, and an understanding of reciprocated sentiments [32,50,54,55,56]. Cognitive-based trust is a character-based approach about the character of a leader. Dirks and Ferrin [32] observed that “trust-related concerns about a leader’s character are important because the leader may have authority to make decisions that have a significant impact on a follower and the follower’s ability to achieve his or her goals (e.g., promotions, pay, work assignments, layoffs)” (p. 612).

Moreover, an employee’s inclination to trust a leader is affected by the actions and character of that leader [57]. Authentic leaders are those who show authenticity and can enhance respect, dignity, integrity, and trust among followers [24]. Authentic leadership is likely to show to subordinates that the leader is concerned about their welfare through the leader’s lack of bias in considering all relevant information before reaching any decisions [39]. In addition, Gardner, Avolio, Luthans and Walumbwa [31] argued that an authentic leader is aware of their own strengths and weaknesses, can present their true self to other people, assists in constructing trust and cooperation, and nurtures team work among colleagues. Such behavior fosters the development of affective-based trust. 

In addition, an authentic leader can act in accordance to their beliefs and moral values that are compatible with their behavior [16]. From the social-exchange perspective, when followers believe in their leader’s authenticity, competence, and honesty, that leader is signaled as an appropriate partner with whom to engage in a social-exchange relationship, which is the characteristic of cognitive-based trust [50]. Past studies indicated a positive and significant association between leadership and followers’ cognitive-based trust [35,54,58]. As mentioned in the earlier sections, past studies have examined the McAllister [50] model of affective- and cognitive-based trust on the association between transformational or ethical leadership and employees’ job outcomes [35,54]. However, to the best of our knowledge, no study has been conducted to date on the direct effect of authentic leadership on affective- and cognitive-based trust. To fill this gap, we investigated the impact of authentic leadership on followers’ affective- and cognitive-based trust (Figure 1), and hypothesized the following.

**Hypothesis** **2.** Authentic leadership is positively associated with affective-based trust.

**Hypothesis** **3.** Authentic leadership is positively associated with cognitive-based trust.

### 2.4. Affective- and Cognitive-Based Trust, and Employees’ Communal Relationships

Affective-based trust is more interpersonal in nature [33] and indicates an exchange-based perspective that happens when a leader engages in a social-exchange relationship with their subordinates [34]. Most relationships between organizational management and employees originate from exchange-based relationships. In order to strengthen these relationships and make them more durable, much depends upon the leadership to propagate them in communal relationships. Empirical evidence showed that affective trust had a strong influence on followers organizational citizenship behavior when compared to cognitive-based trust [55]. However, no study has examined the effect of affective-and cognitive-based trust on employees’ communal relationships. Both affective- and cognitive-based trust are also crucial determinants of the extent to which employees tend to enhance their individual relationships with theirs coworkers and leaders, which ultimately helps in building communal relationships with them. To fill this gap, we assumed that affective- and cognitive-based trust were positively related with employees’ communal relationships (Figure 1), and hypothesized the following:

**Hypothesis** **4.** Affective-based trust is positively associated with employees’ communal relationships.

**Hypothesis** **5.** Cognitive-based trust is positively associated with employees’ communal relationships.

### 2.5. Mediating Role of Affective- and Cognitive-Based Trust on the Relationship between Authentic Leadership and Employees’ Communal Relationships

We examined subordinate trust (affective- and cognitive-based trust) on leaders as a mediating mechanism through which authentic leadership translates its positive influence on communal employee relationships. Trust has been identified as a potential mediator in past studies [59]; previous research using a social-exchange-based perspective for understanding the effect of authentic leadership has not included a clear measure of this key mediating mechanism. Therefore, this was a pioneering study that tested social exchange as a theoretical background for understanding the effect of authentic leadership by integrating affective- and cognitive-based trust as a mediating variable.

As discussed above, authentic leaders are those “who are deeply aware of how they think and behave and are perceived by others as being aware of their own and others values/moral perspectives, knowledge and strengths; aware of the context in which they operate; and who are confident, hopeful, optimistic, resilient, and of high moral character” [30], (p. 4). Moreover, recent empirical evidence reveals the positive effect of authentic leadership on employees’ communal relationship [9]. Past studies have also examined the mediating mechanism of trust in the effect of authentic leadership on followers’ workplace attitudes and behaviors [25,51,52,53]. As discussed above, all mentioned studies have used trust as a unidimensional construct. Focusing on the uniqueness of the McAllister [50] model of affective- and cognitive-based trust, we assumed that trust mediated the relationship between authentic leadership and employees’ communal relationships for several theoretical reasons. First, authentic leaders may enhance subordinates’ affective-based trust by inculcating a sense of obligation, which, in turn, may stimulate subordinates’ sense of a communal relationship. Second, subordinates’ cognitive-based trust is grounded on an authentic leader’s character and positive attributes that helps in reducing the perceived risk in their relationships. In turn, subordinates are more willing to engage in communal relationships. Therefore, on the basis of the aforementioned discussions, we suggested that affective- and cognitive-based trust positively mediates the relationship between authentic leadership and employees’ communal relationships (Figure 1), and posited the following:

**Hypothesis** **6.** Affective-based trust has a positive mediating effect on the relationship between authentic-leadership behaviors and employees’ communal relationships.

**Hypothesis** **7.** Cognitive-based trust has a positive mediating effect on the relationship between authentic-leadership behaviors and employees’ communal relationships.

## 3. Materials and Methods

We adopted a cross-sectional study design and collected data from 200 employees working in different private-sector banking organizations in the city of Peshawar of Pakistan. We highlighted the study’s significance to every bank manager and motivated staff members to participate. After the formal approval of managers, the self-administered questionnaire was distributed among all bank employees, ensuring the confidentiality of the respondents’ responses.

Through a convenient sampling technique, we circulated 300 questionnaires, but received 200 completed questionnaires. Table 1 indicates that out of the 200 total respondents, the majority (143; 71%) were male, and 57 (29%) were female. Most of the respondents (104; 52%) were 21–30 years old, 79 (40%) respondents were 31–40 years old, and the remaining 17 (8%) were 41–50 years old. In addition, the majority (120; 60%) of respondents were married, and 80 (40%) were unmarried; 53 (26%) had a bachelor’s degree, 118 (59%) had a master’s degree, and the remaining 29 (15%) had an MPhil or above level of education. Further, the majority (108; 54%) of respondents had 1–5 years of work experience, 50 (25%) had 6–10 years of experience, 21 (11%) had 11–15 years of experience, and the remaining 12 (6%) had 16–20 years of experience. Lastly, out of the total respondents, only 49 (25%) worked as a bank manager, and the majority (151; 75%) worked as banking staff members.

### 3.1. Measurement

We used a 5-point Likert scale, ranging from strongly disagree to strongly agree, to measure all scales used in the study.

### 3.2. Authentic Leadership

The authentic-leadership scale was measured by using a self-reported scale developed by Walumbwa, Avolio [16]. The scale comprised of 16 items, and sample items included “My manager seeks feedback to improve interactions with others,” “My manager admits mistakes when they are made,” and “My manager demonstrates beliefs that are consistent with actions”. The alpha was 0.873.

### 3.3. Communal Relationships

The communal-relationships scale developed by Clark, Oullette [60] was adopted for the study. The scale comprised 14 items. Example items included “When making a decision, I take other people’s needs and feelings into account” and “I expect people I know to be responsive to my needs and feelings”. The alpha was 0.795.

### 3.4. Affective- and Cognitive-Based Trust

Affective- and cognitive-based trust were measured by using a self-reported scale developed by McAllister (1995). The scale of affective-based trust comprised five items, and cognitive-based trust comprised six items. Sample items of affective-based trust included “My manager and I have a sharing relationship. We can both freely share our ideas, feelings, and hopes” and “If I shared my problems with my manager, I know he would respond constructively and caringly”. The alpha of the affective-based-trust scale was 0.754. Sample items of cognitive-based trust included “My manager approaches his/her job with professionalism and dedication” and “I can rely on my manager not to make my job more difficult by careless work”. The alpha of the cognitive-based-trust scale was 0.726.

## 4. Results 

### 4.1. Descriptive Statistics

The main characteristics of the sample, including means, standard deviations, and variable correlations, are shown in Table 2. Correlation between authentic leadership and communal relationships (*r* = 0.339, *p* < 0.01) was found to be positive and significant, as expected; likewise, the correlation between authentic leadership and affective-based trust (*r* = 0.511, *p* < 0.01) was also found to be positive and significant. The correlation between authentic leadership and cognitive-based trust was *r* = 0.382, *p* < 0.01. In addition, correlation between communal relationship and affective-based trust (*r* = 0.410, *p* < 0.01) was found significant, as expected. Communal-relationship correlation with cognitive-based trust (*r* = 0.347, *p* < 0.01) was also found to be positive and significant, as expected. Finally, correlation between affective- and cognitive-based trust (*r* = 0.566, *p* < 0.01) was also found significant.

### 4.2. Confirmatory-Factor Analysis (CFA) for Authentic Leadership, Communal Relationships, and Affective- and Cognitive-Based Trust 

To examine the convergence and discriminant validity of the scales, CFA was performed by using SPSS Amos version 20. First, authentic leadership, communal relationships, affective- and cognitive-based trust were compared in a hypothesized four-factor model (Model 1) with three other models. Second, communal relationships and affective-based-trust items were combined into a new single factor in the three-factor model (Model 2). Third, communal relationships, and affective- and cognitive-based-trust items were combined into a new single factor in a two-factor model (Model 3). Finally, we loaded all items of the studied variables (authentic leadership, affective- and cognitive-based trust, and communal relationships together into a new single factor in a one-factor model (Model 4). Confirmatory-factor analysis with maximum-likelihood estimation was performed for all three proposed models. Factor-loading for each component was found positive and significant, and indicated good convergence validity. Average extracted variance of all proposed variables was checked, and the square root of every average variance extracted (AVE) was found to be greater than all variable coefficients [61]. Results shown in Table 3 indicate a good model fit for the hypothesized three-factor model (Model 1; chi square/degree of freedom (CMIN/DF) = 1.366, comparative fit index (CFI) = 0.918, incremental fit indices (IFI) = 0.920, Tucker–Lewis Index (TLI) = 0.907, root mean square error of approximation (RMSEA) = 0.043) compared to other alternative models.

### 4.3. Regression Analysis of Authentic Leadership, Affective- and Cognitive-Based Trust, and Communal Relationships

Multiple linear regression was performed to examine the main hypotheses of the study. Results presented in Table 4 indicate the influence of the independent variable (authentic leadership) and control variables (gender and age) on the dependent variable (communal relationships).

Results presented in Table 4 indicate the positive association of authentic leadership with communal relationships (β = 0.344, *p* < 0.0001), supporting Hypothesis 1. Hypothesis 2 predicted the positive relationship between authentic leadership with affective-based trust. Results revealed that authentic leadership had a positive association with affective-based trust (β = 0.553, *p* < 0.0001); hence, Hypothesis 2 was also fully supported. Hypothesis 3 predicted the positive association between authentic leadership with cognitive-based trust. Results revealed that authentic leadership had a positive association with cognitive-based trust (β = 0.520, *p* < 0.0001); hence, Hypothesis 3 was also fully supported. Hypothesis 4 predicted the positive association between affective-based trust with communal relationships. Results indicated that affective-based trust had a positive and significant association with communal relationships (β = 0.386, *p* < 0.0001); hence, Hypothesis 4 was supported. Similarly, Hypothesis 5 predicted a positive association between cognitive-based trust with communal relationships. Results indicated that cognitive-based trust had a positive and significant association with communal relationships (β = 0.259, *p* < 0.0001); hence, Hypothesis 5 was also fully supported.

### 4.4. Mediation Analysis

In the current study, the process program for SPSS developed by Hayes [62] was used to analyze the mediating hypotheses. For this, we selected Model 4 from the Hayes templates to find the direct influence of authentic leadership on communal relationships, as well as the mediating role of affective- and cognitive-based trust on the association between authentic leadership and communal relationships. Moreover, a 95% correct bias confidence interval with 5000 bootstrapping-procedure sample estimates was selected. 

In Hypothesis 6, we hypothesized that affective-based trust had a positive mediating influence on the association between authentic leadership and communal relationships. The results (Table 5) showed that affective-based trust mediated the association between authentic leadership and communal relationships (β = 0.177, *p* < 0.02), supporting Hypothesis 6.

Similarly, in Hypothesis 7, it was hypothesized that cognitive-based trust had a positive mediating influence on the association between authentic leadership and communal relationships. The results shown in Table 6 revealed that cognitive-based trust mediated the association between authentic leadership and communal relationships (β = 0.177, *p* < 0.02), supporting Hypothesis 7.

## 5. Discussion

The present study explored the direct effect of authenticity on employees’ communal relationships as well as the indirect effect of an authentic leadership on employees’ communal relationships through affective- and cognitive-based trust. 

This study indicated the vital role of authentic leadership in affecting employees’ communal relationships. As discussed in the literature section, authentic leadership has attracted researchers’ attention due to its positive role in affecting employees and organizational-goal achievements [16,17,18], calling for more empirical work [7,8,19]. We filled this gap by investigating the impact of authentic leadership on communal relationships among employees working in the banking sector of Pakistan. Moreover, in line with past studies [9], this study revealed the positive role of authentic leadership in affecting employees’ communal relationships, supporting Hypothesis 1. 

On the basis of relational model theory by Fiske [28] and its theoretical component, specifically the communal-sharing mental schema, we argue that, when employees perceive their leader’s behavior as authentic, they display more concern for their work and take care of organizational fame, which aids the organization in accomplishing its goals. By adding relational-model theory, this study makes an important theoretical contribution to the literature in the field of psychology.

In addition, the current study significantly contributes to the existing literature related to authentic leadership and trust by giving a more comprehensive understanding of the mediating role played by trust on the association between authentic leadership and employees’ workplace-relationship outcomes than past studies, which typically hypothesized trust as a one-dimensional construct. Dirks and Ferrin [32], in their meta-analytic review, urged researchers to consider multiple constructs of trust, including affective- and cognitive-based trust, and “attempt to distinguish between the processes involved” (p. 623), but very few studies have followed this call [35,36,37]. We filled this breach and explored the indirect effect of authentic leadership on employees’ communal relationships through affective- and cognitive-based trust. In agreement with our initial expectations, authentic leadership was found to be positively associated with affective- and cognitive-based trust. In addition, affective- and cognitive-based trust both positively mediated the relationship between authentic leadership and employees’ communal relationships. From a social-exchange perspective, our findings have also revealed that trust stimulates positive workplace-relationship outcomes, which is important; this is pioneering, as it examined both affective- and cognitive-based trust as a mediating mechanism on exploring the relationship between authentic leadership with employees’ communal relationships, making a new contribution to the authentic-leadership literature. 

### Practical Implications, Limitations, and Future Research 

The current study has confirmed that leadership plays an important role in influencing employee attitudes and behaviors within organizations. The results of the study suggested that an organization needs to pay specific attention to authentic leadership and to enhance follower workplace-relationship outcomes, such as employees’ communal relationships. To gain the full benefits of authentic leadership, organizations should consider integrating components of authentic leadership into their development, appraisal, and selection processes. For instance, an organization may give more importance to training both leaders and subordinates to advance their concerns with regard to workplace issues. This results in more authentic behavior by leaders, as well as enhancing the receptivity of subordinates to such behaviors. The current study also revealed the vital role of the affective- and cognitive-based-trust mediating mechanism underlying the relationship between authentic leadership and communal relationships. This study suggests that leaders should rationally consider approaches they utilize to influence their subordinates’ behaviors in the workplace. Specifically, our study suggests that authentic leadership may be used as an instrument by managers to influence employees’ communal relationships through the development of trust.

This study also had some limitations. First, we only focused on the private banking sector. Hence, it is suggested that the study should be extended to other work settings to further develop the understanding of relationships between the studied variables. Second, common-method bias was also a limitation. Furthermore, studies should be expanded to other departments and groups as well. Third, the current study should be replicated in other countries to increase the generalizability of our findings. Future studies are encouraged to consider other organizational variables, such as work engagement and well-being, while focusing on authentic leadership and trust.

## 6. Conclusions

This study contributes to the existing literature by examining trust-based mechanisms on the relationship between authentic leadership and employees’ communal relationships. In addition, it also makes an important contribution by clearly presenting how authentic leadership influences employees’ communal relationships through affective- and cognitive-based trust. Our results revealed that both cognitive- and affective-based trust positively mediate the relationship between authentic leadership and employees’ communal relationships. We hope that the present study will encourage future researchers to examine the trust-based mechanisms by which authentic leadership influences employee work outcomes.

## Figures and Tables

**Table 1:** Demographic variable results.

Demographic Variables	Frequencies	Sample Percentage
**Respondent Gender**		
Male	143	71%
Female	57	29%
**Respondent Age**		
21–30 years	104	52%
31–40 years	79	40%
41–50 years	17	8%
**Respondent Marital Status**		
Married	120	60%
Unmarried	80	40%
**Respondent Education**		
Bachelor’s degree	53	26%
Master degree	118	59%
MPhil and above	29	15%
**Respondent Work Experience**		
1–5 years	108	54%
6–10 years	50	25%
11–15 years	21	11%
16–20 years	12	6%
**Respondent Job title**		
Manager	49	25%
Staff member	151	75%
**Total**	200	100

**Table 2:** Descriptive statistics, mean, standard deviation (SD), and correlation of variables.

Heading Column	Mean	SD	1	2	3	4
Authentic leadership	3.573	0.55903	1			
Communal relationship	3.6014	0.56811	0.339 **	1		
Affective-based trust	4.0130	0.60420	0.511 **	0.410 **	1	
Cognitive-based trust	3.9183	0.76129	0.382 **	0.347 **	0.566 **	1

Note: *N* = 200; ** *p* < 0.01

**Table 3:** Confirmatory-factor-analysis results for authentic leadership, affective- and cognitive-based trust, and communal relationships.

Measurement Models	CMIN/DF	CFI	IFI	TLI	RMSEA
M1. Hypothesized four-factor model.	1.366	0.918	0.920	0.907	0.043
M2. Three-factor model: affective-based trust and communal relationships were merged.	1.550	0.876	0.880	0.869	0.053
M3. Two-factor model: Affective- and cognitive-based trust, and communal relationships were merged.	1.636	0.857	0.861	0.838	0.057
M4. One-factor model, authentic leadership, affective- and cognitive-based trust, and communal relationships were merged.	1.830	0.819	0.825	0.789	0.065

Abbreviations: CMIN/DF, chi-square/degree of freedom; CFI, comparative fit index; IFI, incremental fit indices: TLI, Tucker–Lewis Index; RMSEA, root mean square error of approximation.

**Table 4:** Regression analysis of authentic leadership, affective- and cognitive-based trust, and communal relationships.

Variables	Affective-Based Trust	Cognitive-Based Trust	Communal Relationships
Constant			
Gender	128	0.348 **	0.119
Age	−0.023	−0.009	0.044
Authentic leadership	0.553 ***	0.520 ***	0.344 ***
Affective-based trust			0.386 ***
Cognitive-based trust			0.259 ***
*R^2^*	0.262	0.146	0.115
∆*R^2^*	0.258	0.142	0.110
F	70.152 ***	33.863 ***	25.631 ***

Note: *N* = 200; ** *p* < 0.01; *** *p* < 0.0001.

**Table 5:** Mediation coefficient and bootstrapping.

Testing Paths	Unstandardized Coefficient		T	Sig	Bootstrapping	
	Standard Coefficient Error				LLCI ULCI	
IV→M (a)	0.553	0.066	8.376	0.0001	0.423	0.683
M→DV (b)	0.302	0.070	4.300	0.0001	0.163	0.440
IV→M→DV (c’)	0.177	0.076	2.331	0.02	0.028	0.327
IV→DV (c)	0.344	0.068	5.063	0.0001	0.210	0.478
Indirect effects	0.167	0.048			0.083	0.327

Note: IV (Authentic leadership), MV (affective-based trust), DV (communal relationships).

**Table 6:** Mediation coefficient and bootstrapping.

Testing Paths	Unstandardized Coefficient		T	Sig	Bootstrapping	
	Standard Coefficient Error				LLCI ULCI	
IV→M (a)	0.520	0.089	5.819	0.0001	0.344	0.697
M→DV (b)	0.190	0.052	3.632	0.0001	0.087	0.294
IV→M→DV (c’)	0.245	0.071	3.432	0.001	0.104	0.386
IV→DV (c)	0.344	0.068	5.063	0.0001	0.210	0.478
Indirect effects	0.099	0.035			0.042	0.182
